# Physical activity changes and related factors in chronic heart failure patients during the postdischarge transition period: a longitudinal study

**DOI:** 10.1186/s12872-024-03881-4

**Published:** 2024-04-30

**Authors:** Yingtong Meng, Tingting Zhang, Xiaohua Ge, Qingru Zheng, Tienan Feng

**Affiliations:** 1 Cardiology Department II ward I, Xinhua Hospital, School of Medicine, Shanghai Jiao Tong University, Shanghai, 200092 People’s Republic of China; 2 Department of Nursing, Xinhua Hospital, School of Medicine, Shanghai Jiao Tong University, Shanghai, 200092 People’s Republic of China; 3 Department of Intensive Care Medicine, The Sixth People’s Hospital, Shanghai Jiao Tong University, Shanghai, 200233 People’s Republic of China; 4 Clinical Research Institute, Shanghai Jiao Tong University School of Medicine, Shanghai, 200025 People’s Republic of China

**Keywords:** Chronic heart failure, Physical activity, Longitudinal study, CHF-related symptoms, Kinesiophobia

## Abstract

**Background:**

Physical activity (PA) is essential and effective for chronic heart failure (CHF) patients. A greater understanding of the longitudinal change in PA and its influencing factors during the postdischarge transition period may help create interventions for improving PA. The aims of this study were (1) to compare the change in PA, (2) to examine the influencing factors of PA change, and (3) to verify the mediating pathways between influencing factors and PA during the postdischarge transition period in CHF patients.

**Methods:**

A total of 209 CHF patients were recruited using a longitudinal study design. The Chinese version of the International Physical Activity Questionnaire (IPAQ), Patient-reported Outcome Measure for CHF (CHF-PRO), and the Chinese version of the Tampa Scale for Kinesiophobia Heart (TSK-Heart) were used to assess PA, CHF-related symptoms, and kinesiophobia. The IPAQ score was calculated (1) at admission, (2) two weeks after discharge, (3) two months after discharge, and (4) three months after discharge. Two additional questionnaires were collected during admission. Generalized estimating equation (GEE) models were fitted to identify variables associated with PA over time. We followed the STROBE checklist for reporting the study.

**Results:**

The PA scores at the four follow-up visits were 1039.50 (346.50-1953.00) (baseline/T1), 630.00 (1.00-1260.00) (T2), 693.00 (1-1323.00) (T3) and 693.00 (160.88–1386.00) (T4). The PA of CHF patients decreased unevenly, with the lowest level occurring two weeks after discharge, and gradually improving at two and three months after discharge. CHF-related symptoms and kinesiophobia were significantly associated with changes in PA over time. Compared with before hospitalization, an increase in CHF-related symptoms at two weeks and two months after discharge was significantly associated with decreased PA. According to our path analysis, CHF-related symptoms were positively and directly associated with kinesiophobia, and kinesiophobia was negatively and directly related to PA. Moreover, CHF-related symptoms are indirectly related to PA through kinesiophobia.

**Conclusion:**

PA changed during the postdischarge transition period and was associated with CHF-related symptoms and kinesiophobia in CHF patients. Reducing CHF-related symptoms helps improve kinesiophobia in CHF patients. In addition, the reduction in CHF-related symptoms led to an increase in PA through the improvement of kinesiophobia.

**Trial Registration:**

The study was registered in the Chinese Clinical Trial Registry (11/10/2022 ChiCTR2200064561 retrospectively registered).

## Background

Currently, more than 64 million people in the world suffer from chronic heart failure (CHF) [1], and the number in China was 8.9 million in 2021 [2]. Patients with CHF continuously suffer from severe CHF-related symptoms such as dyspnea, orthopnea, and lower limb swelling, whose one-year mortality and readmission rates are as high as 17% and 44%, respectively [3]. Historically, patients with CHF were assumed to be at risk of exercise and were commonly discouraged from participating in physical activity (PA). In contrast to these concerns, studies have shown that adequate PA improves not only CHF patients’ quality of life but also physical function, CHF-related symptoms, and readmission rate [4]. However, exercise-based cardiac rehabilitation (CR) remains underused. Only 4% of CHF patients actively participate in exercise-based CR after discharge [5]. The postdischarge transition period is generally defined as three months after discharge [6]. The unplanned readmission rate is the highest, and patients’ physical and psychological states and behaviors change dynamically during this period [7]. This is a crucial period for CHF patients. Thus, effective transitional care programs to promote regular PA for CHF patients are urgently needed. Information on PA change trajectories and influencing factors can lead to the development of customized and holistic measures to enhance PA during different recovery periods and facilitate the rational allocation of care resources in patients with CHF.

In previous studies, several sociodemographic, medical risk, and psychological factors have been shown to be associated with PA in CHF patients. The PA concentration is generally lower in older patients [8]. The effect of sex and education level on PA in CHF patients is unclear [9, 10]. Pathological changes in the heart are known to lead to a series of distressful symptoms, such as dyspnea, fatigue, and lower limb ooedema, in patients with CHF. All of these CHF-related symptoms lead to a decrease in the patient’s exercise endurance and a reduction in exercise [11]. In addition, psychological disorders such as kinesiophobia may affect patients’ PA [12]. Kinesiophobia is an excessive and irrational fear of PA or exercise due to the fear of harm or reinjury. The presence of kinesiophobia modulates individuals’ perceptions and responses to illness and affects functional levels and participation [13]. In addition, kinesiophobia has been associated not only with cardiovascular diseases but also with other conditions, such as musculoskeletal pain disorders, pulmonary diseases, and falls [14]. Kinesiophobia is a well-known independent barrier for patients performing PA [12]. However, most of these influencing factors were identified through cross-sectional studies. Compared to cross-sectional studies, longitudinal studies focus on tracking time, which reflects changes and predictors of each change trajectory. Therefore, there is an urgent need for a longitudinal study to confirm the long-term and dynamic effects of demographic factors, CHD-related syndromes, and kinesiophobia on the trajectory of change in PA in CHF patients during the postdischarge transition period.

In addition, a previous study showed that CHF-related symptoms were significantly correlated with kinesiophobia, and the relief of CHF-related symptoms reduced kinesiophobia in CHF patients [12]. Moreover, the negative impact of CHF-related symptoms on PA is undoubted. Similarly, the fear avoidance (FA) model also proposes that physical negative experiences may indirectly affect patients’ physical movements through kinesiophobia [15]. However, the interaction path between CHF-related symptoms, kinesiophobia, and PA in CHF patients during the postdischarge transition period has yet to be fully elucidated. Therefore, path analysis is needed to verify the action mechanism of the above factors on PA.

In summary, cross-sectional studies have shown that CHF patients have poor PA during the postdischarge transition period. CHF-related symptoms and kinesiophobia negatively impact patients’ PA. However, few longitudinal studies have investigated the changes in PA over time and the long-term and dynamic impacts of sociodemographic, medical risk, and psychological factors on PA in CHF patients. Furthermore, the mediating role of kinesiophobia between CHF-related symptoms and PA in CHF patients has not yet been determined in the database. Hence, the present study used a longitudinal approach to describe the changes in PA trajectories during the postdischarge transition period; explore the effects of demographic factors, disease characteristics, CHF-related symptoms, and kinesiophobia on PA; and examine the indirect (mediating) effects of kinesiophobia between CHF-related symptoms and PA in CHF patients during the postdischarge transition period.

## Methods

### Aims

The aims of the study were (1) to describe the changes in PA during the postdischarge transition period; (2) to explore the effects of demographic characteristics, disease characteristics, CHF-related symptoms, and kinesiophobia on PA; and (3) to test the indirect (mediating) effect of CHF-related symptoms on PA through kinesiophobia among CHF patients during the postdischarge transition period.

### Study design and objective

This study followed a longitudinal design guided by the Strengthening the Reporting of Observational Studies in Epidemiology (STROBE) checklist.

### Setting and participants

This study enrolled CHF patients from a cardiovascular medicine ward at the study hospital via a convenience sample between December 2020 and November 2021. The inclusion criteria were: (1) age ≥18 years old; (2) diagnosis of CHF based on the 2018 Clinical Guidelines for the Diagnosis and Treatment of HF in China [16]: symptoms (e.g., dyspnea, fatigue), signs (e.g., elevated jugular venous pressure, peripheral oedema), or laboratory/imaging evidence (e.g., abnormal cardiac structure and cardiac systolic and diastolic function on echocardiography, N-terminal pro-B-type natriuretic peptide (NT-proBNP) ≥ 125 ng/L or B-type natriuretic peptide (BNP) ≥ 35 ng/L); (3) New York Heart Association (NYHA) Classification grading II – III; (4) The risk stratification of cardiac events during exercise was low to moderate according to the exercise-related risk stratification [17]; and (5) willingness and ability to complete questionnaires. The exclusion criteria were as follows: (1) with acute HF; (2) impacted by complications of cardiovascular diseases (e.g., high-risk unstable angina, uncontrolled arrhythmia, severe or symptomatic aortic stenosis, hypertrophic obstructive cardiomyopathy, recent thrombosis, severe pulmonary hypertension, or uncontrolled hypertension); (3) diagnosed with diseases associated with the muscles, bones, joints, or nervous system that severely impacted patients’ ability to participate in physical activities; or (4) had a history of dementia, mental illness, or intellectual disability.

The study included 205 participants. The GPower version 3.1 was used for the power analysis [18]. Based on study by Cohen et al., the effect sizes in case multiple means (multiple groups) are present have been set at 0.10, 0.25, and 0.40, which represent small, medium, or large effect sizes respectively [18]. We set the effect size = 0.1/0.25/0.4, α err prob = 0.05, the number of predictors = 13, and the total sample size = 205 [19]. The result showed that the power (1- β err prob) was 0.839/0.999/0.999, suggesting that the average statistical power of this study was good (1-β > 0.80).

### Recruitment and data collection

Voluntary informed consent was obtained from each participant at admission by three investigators (Meng, Zheng, and Zhang) and written informed consent was obtained from all participants in the study. Patient demographic and disease characteristic data were collected through the hospital information system and through communication with patients or their family members. Structured questionnaires were used to assess PA, CHF-related symptoms, kinesiophobia, demographic information, and disease characteristics. The PA questionnaire was administered (1) at admission, (2) two weeks after discharge, (3) two months after discharge, and (4) three months after discharge. Three other questionnaires were collected during admission. Patients were required to return the questionnaires upon completion. The first collection was conducted face-to-face, and what patients completed in the PA section was their activity one week prior to hospitalization. The patients’ PA was collected via phone for the following three collections (at two weeks, two months, and three months after discharge). If patients provided vague answers, they were repeatedly asked rhetorical questions or had their answers reorganized to clarify their meaning, confirm their accuracy, and ensure our understanding.

### Ethical considerations

This study received ethical approval from the institutional review board of Xinhua Hospital, School of Medicine, Shanghai Jiao Tong University (XHEC-C-2021-108-1). The protocol of this study adhered to the Code of Ethics of the Declaration of Helsinki. Written Informed consent was obtained from all participants in the study. All participants took part voluntarily and could withdraw from the study at any time.

### Measurements

#### General information

A self-designed general information questionnaire was used to collect demographic and disease characteristics. The information collected included sex, age, admission methods, marital status, education level, living area, monthly family income, occupational status, whether the patient was the first diagnosis of CHF, New York Heart Association (NYHA) classification, left ventricular ejection fraction (LVEF), and NT-pro BNP level.

#### PA

The Chinese version of the International Physical Activity Questionnaire (IPAQ) was used to assess the PA of CHF patients. The International PA Evaluation Collaborative Group designed the questionnaire to collect detailed information about household and yard work activities, occupational activity, self-powered transport, leisure-time PA, and sedentary activity [20]. Each activity has a fixed energy expenditure value expressed by (metabolic equivalent tasks (METs). During the evaluations, patients were asked to recall their physical activity type, duration, and frequency during the previous week. PA was assigned an energy value, and MET-min/week was calculated as MET * PA duration (min) * frequency. The Chinese version of IPAQ had good internal reliability and test-retest stability (total ICC = 0.84), and had a significantly correlated between pedometer (*r* = 0.280-0.561) [21]. The questionnaire’s total ICC was calculated to be equal to 0.879 in this study.

### CHF-related symptoms

The symptoms of the CHF patients were assessed by the physiological domain of the Patient-reported Outcome Measure for CHF (CHF-PRO). The scale was designed by Xue et al. [22] The CHF-PRO includes four domains, 12 dimensions, and 57 items. The symptoms that this scale can assess include (1) somatic symptoms (such as dyspnea, orthopnea, and lower limb swelling), (2) appetite and sleep, and (3) independence. Each question was scored on a five-point Likert scale, and the total score ranged from 0∼100 points after standardization. The scores are inversely proportional to symptom severity. The Cronbach’s α was 0.913 [22]. In this study, the Cronbach’s α was 0.904.

### Kinesiophobia

The Chinese version of the Tampa Scale for Kinesiophobia Heart (TSK-Heart) was used to evaluate kinesiophobia. This questionnaire was adapted from Bäck et al.[23]. The scale consists of 17 items and four dimensions: (1) perceived cardiac risk, (2) avoidance of exercise, (3) fear of injury, and (4) decreased self-function. Each question was scored on a four-point Likert scale, and the total score ranged from 17∼68 points. Higher scores indicate a greater fear of exercising. The Cronbach’s α was equal to 0.895 [24]. In this study, the Cronbach’s α was 0.812.

### Data analysis

The data were analyzed using IBM SPSS version 25. Descriptive statistics were used to analyze the baseline characteristics. For continuous variables, Kolmogorov–Smirnov analysis was applied to test the distribution. Normally distributed data were presented as the mean ± SD; if not normally distributed, the data were presented as the median (interquartile range). Categorical values awere expressed as frequencies (percentages). The Kolmogorov-Smirnov test of the PA revealed a significant difference (*P* < 0.05), which indicated a nonnormal distribution.

This study used a generalized estimating equation (GEE) to analyze the changes in PA levels during the postdischarge transition period and the factors associated with the changes in PA levels over time. GEE was used in our study because, unlike repeated-measures ANOVA methods, the GEE model has the advantage that it does not require the outcome variable to have a normal distribution and constant variance across time points or constant correlation between any two time points. GEE did not exclude subjects with missing observations at one or more time points. In particular, GEE focuses on overall average response changes over time and covariate impacts on these changes [25]. The application of GEE in our study modeled the change in PA level as a function of time, sex, age, admission methods, etc., as well as CHF-related symptoms and kinesiophobia. The GEE model was used with the identity link function and the autoregressive (AR(1)) correlation structure. An identity link function is used because PA is a continuous outcome. The AR(1) correlation structure is specified because PA levels correlate more when assessments are performed more closely over time [25]. A *p* value < 0.05 was considered to indicate statistical significance.

Path analysis was performed with SPSS Amos version 23 to test for the mediating effect of kinesiophobia between CHF-related symptoms and PA. An acceptable goodness of fit of the model was determined by the following factors: the ratio of the chi-square value to the degree of freedom was less than 5, the goodness-of-fit index was greater than 0.90, the comparative fit index was greater than 0.90, the Tucker–Lewis index was greater than 0.90, and the root-mean-square error approximation was less than 0.08.

## Results

### Recruitment

**Fig. 1:**
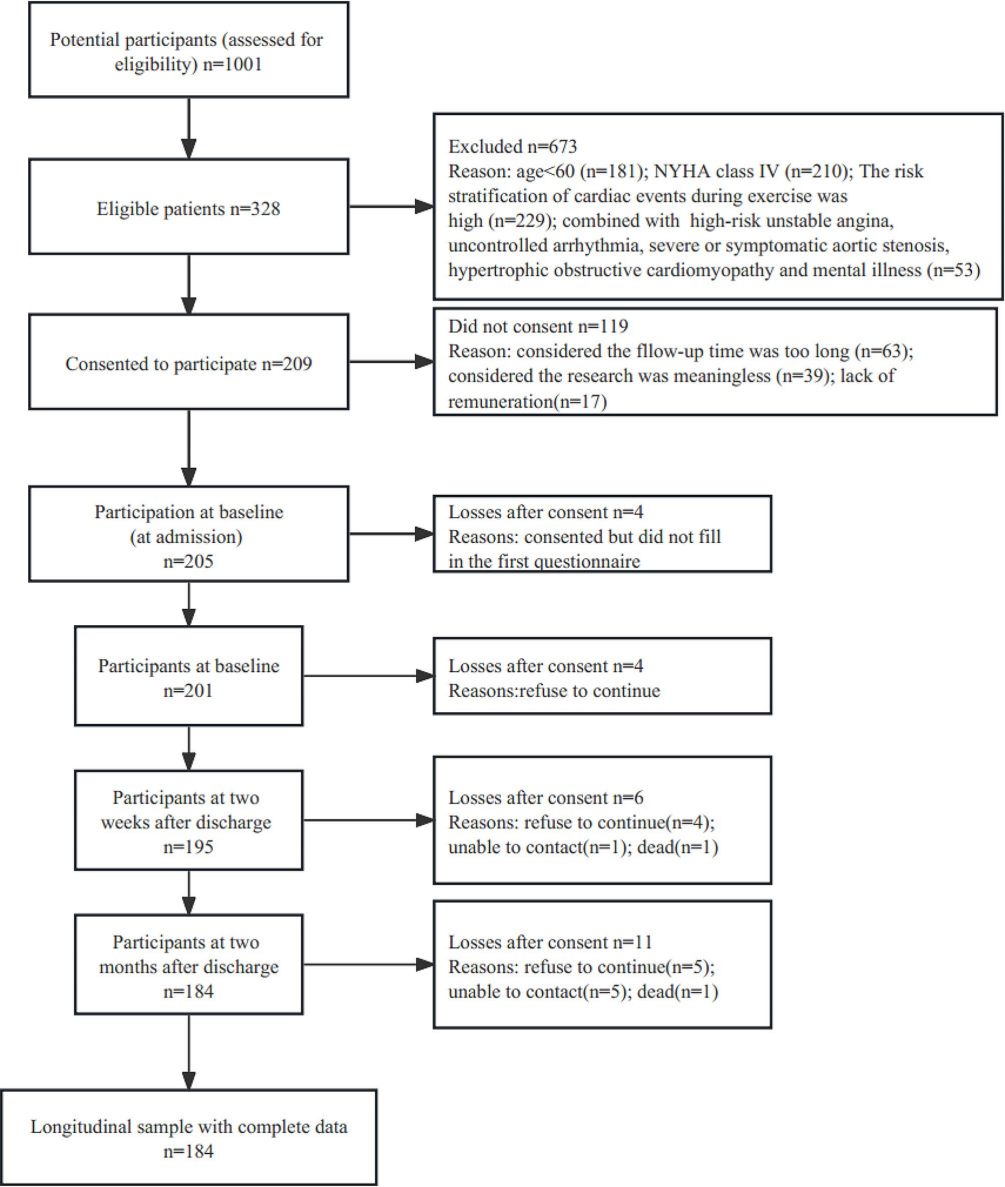
The recruitment process and the protocol used in this study

Figure 1 showed the recruitment process and the protocol followed in this study. Of the 328 eligible CHF patients, 205 (62.5%) answered the baseline questionnaire, and 184 (89.8%) completed the study. There were no significant differences in the baseline characteristics between the patients included in the final sample and those lost to follow-up (*p* > 0.05).

### Sample characteristics

Among 184 CHF patients, a majority were male (63.0%), 60∼74 years old (75.0%), married (81.0%), lived in city (86.2%), junior high school educational level (40.0%), family monthly incomes >10,000 (45.1%), mental work (43.5%), not first diagnosis of CHF (53.3%), NYHA Classification Grade III (55.4%), LVEF ≥ 50% (46.2%), NT-pro BNP 1001∼5000 (64.7%).

### Changes in PA during the postdischarge transition period

**Fig. 2:**
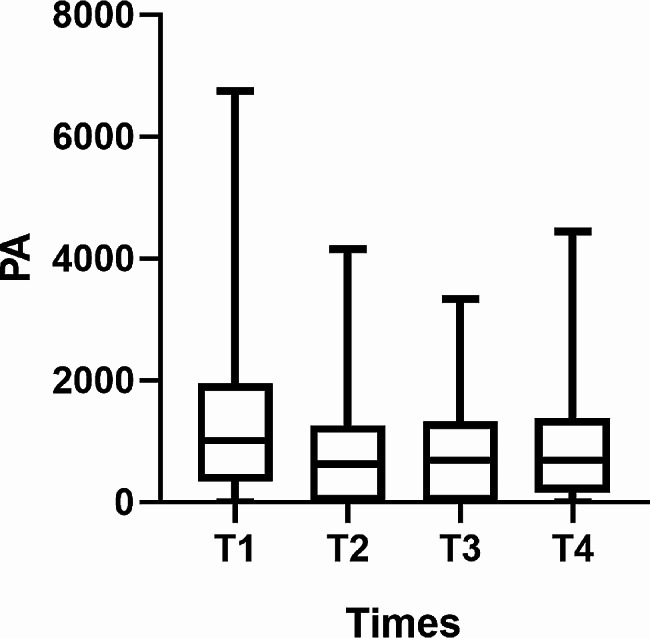
Changes in PA over time

The PA scores at the four follow-up visits were 1039.50 (346.50-1953.00) (baseline/T1), 630.00 (1.00-1260.00) (T2), 693.00 (1.00-1323.00) (T3) and 693.00 (160.88-1386.00) (T4). GEE analysis indicated that PA decreased overall during the postdischarge transition period (*Wald χ*^*2*^ = 66.903, *P*<0.001). Further paired comparison of PA at each time point showed PA at T2 significantly decreased compared to T1(*Z*=-6.967, *P*<0.001); The change in physical activity at T3 was not significant compared to T2(*Z*=-0.856, *P* = 0.395); PA at T4 significantly increased compared to T3(*Z*=-4.215, *P*<0.001; PA at T4 significantly decreased compared to T1 (*Z*=-3.010, *P* = 0.003) (see Fig. 2).

### Factors associated with PA during the postdischarge transition period

Table 1 showed the factors that correlated with PA based on the univariate model of GEE. The results showed whether the patient was the first diagnosis of CHF, NYHA classification grade, CHF-related symptoms, and kinesiophobia were significantly correlated with PA during the postdischarge transition period. These four factors and time points were included in the multivariable model of GEE analysis.

**Table 1:** Participant characteristics and factor analysis of PA according to the univariate GEE model (*n* = 184)

	n/mean±SD	T1	T2	T3	T4	β	SE	95% CI
		IPAQ score	IPAQ score	IPAQ score	IPAQ score			
Sex								
Female	68	810(175.50, 1953.00)	250.50(1.00, 939.00)	315.00(1.00, 1203.75)	315.00(1.00, 1260.00)	0^a^	-	-
Male	116	1181.25(495.00, 1953.00)	693.00(1.00, 1323.00)	963.00(322.875,1396.125)	1072.50(565.875, 1521.00.00)	175.565	177.123	-171.590∼522.721
Age								
60∼75	138	1162.50(490.50, 1968.00)	693.00(1.00, 1323.00)	865.50(297.00, 1374.375)	1046.25(378.00, 1567.125)	0^a^	-	-
76∼80	29	693.00(1.00, 2236.50)	1.00(1.00,693.00)	1.00(1.00, 1023.00)	198.00(1.00, 873.00)	-107.268	287.34	-670.436∼455.900
>80	17	778.50(149.00, 1755.00)	120.00(1.00, 724.50)	487.00(1.00, 1089.00)	495.00(1.00, 828.00)	-394.766	282.543	-948.540∼159.007
Return to work								
Yes	17	1953.00(925.50, 2452.00)	976.50(693.00, 1323.00)	1323.00(693.00, 1389.00)	1323.00(828.00, 2013.75)	0^a^	-	-
No	167	1008.00(318.00, 1638.00)	630.00(1.00, 1260.00)	693.00(1.00, 1323.00)	693.00(135.00, 1354.00)	-694.669	364.889	-1409.837∼20.500
Marital status								
Married	149	1039.50(346.50, 1668.00)	630.00(1.00, 1177.50)	693.00(127.50, 1323.00)	693.00(135.00, 1369.50)	0^a^	-	-
Unmarried, Divorced, or Widowed	35	1260.00(306.00, 2362.50)	693.00(1.00, 1354.50)	855.00(1.00, 1386.00)	616.50(1.00, 1453.50)	669.218	1008.918	-1308.224∼2464.660
Residence								
Urban	159	976.50(318.00, 1953.00)	495.00(1.00, 1204.50)	693.00(1.00, 1323.00)	693.00(135.00, 1386.000)	0^a^	-	-
Rural	25	1260.00(967.50, 2247.00)	976.50(630.00, 1371.50)	693.00(495.00, 1488.00)	1113.00(330.75, 1585.50)	554,669	298.473	-30.328∼1139.666
Education Level								
No formal education	9	1050.00(704.25, 1496.25)	630.00(135.50, 1008.00)	148.50(0.500, 645.75)	346.50(113.00, 924.75)	0^a^	-	-
Primary school	27	1008.00(315.00, 2772.00)	693.00(1.00, 1455.00)	1113.00(1.00, 1569.00)	661.50(1.00, 1521.00)	474.481	304.779	-122.874∼1071.837
Junior high school	68	926.25(246.50, 2016.00)	630.00(1.00, 1260.00)	711.00(1.00, 1323.00)	693.00(34.50,1545.75)	105.377	203.310	-293.102∼503.857
High School/Secondary School	49	1155.00(346.50, 1606.50)	630.00(1.00, 1223.25)	693.00(173.25, 1323.00)	924.00(330.75, 1323.00)	108.180	225.687	-334.157∼550.518
College/Junior college/Bachelor or above	31	1188.00(109.50, 1914.75)	594.00(1.00, 927.00)	693.00(1.00, 1456.50)	68.00(1039.50, 1779.75)	86.506	256.2003	-415.638∼588.649
Underlying disease								
Myocardial disease	98	1172.25(444.375, 2079.00)	693.00(1.00, 1386.00)	873.00(1.00, 1505.25)	888.00(222.75, 1567.13)	0^a^	-	-
Abnormal cardiac load	23	1188.00(693.00, 2016.00)	630(1.00, 1204.50)	630(1.00, 1323.00)	693(1.00, 1669.50)	-113.813	236.291	-576.93∼349.308
Arrhythmia	63	792.00(315.00, 1386.00)	270.00(1.00, 1546.20)	630.00(1.00, 1494.60)	693.00(135.00, 1323.00)	-354.926	185.971	-719.422∼9.570
**First diagnosis of CHF?**								
Yes	86	1260.00(693.00, 2095.50)	693.00(1.00, 1375.875)	976.50(282.75, 1466.25)	1072.50(443.625,1654.875)	0^a^	-	-
**No**	**98**	**786.00(120.00,1323.00)**	**976.50(282.75, 1466.25)**	**630.00(1.00, 1188.375)**	**668.25(1.00, 1323.00)**	**-479/102**	**169.415**	-811.149~-147.054*
**NYHA Classification Grading**								
II	85	1188.00(693.00,2145.00)	826.50(420.00, 1455.00)	1053.00(630.00, 1521.00)	1323.00(661.50, 1732.00)	Reference		
**III**	**102**	**919.50(123.75, 1390.50)**	**255.00(1.00, 976.50)**	**423.00(1.00, 1122.00)**	**630.00(1.00, 1225.00)**	**-1842.826**	**499.288**	-2821.413~-864.239*
LVEF (%)								
< 40	44	1023.75(307.75, 1579.125)	693.00(1.00, 1078.875)	693.00(1.00, 1350.00)	693.00(34.50, 1365.75)	0^a^	-	-
40∼49	55	1050.00(501.00, 1557.00)	693.00(1.00, 1404.00)	729.00(315.00, 1420.00)	963.00(297.00, 1732.50)	233.524	207.874	-173.901∼640.949
≥ 50	85	1008.00(315.00, 2016.00)	630.00(1.00, 1232.25)	693.00(1.00, 1323.00)	693.00(135.00, 1338.75)	261.574	203.231	-136.752∼659.899
NT-pro BNP								
125∼1000	48	1044.75(346.50, 2016.00)	693.00(30.75, 1281.00)	816.00(30.75, 1487,25)	1201.50(528.75, 1810.125)	0^a^	-	-
1001∼5000	119	1008.00(340.00, 1953.00)	693.00(1.00, 1232.00)	693.00(1.00, 1323.00)	693.00(148.50, 1359.00)	11.427	178.840	-339.092∼361.947
>5000	17	1137.00(603.75, 1480.50)	1.00(1.00, 945.00)	297.00(1.00, 402.75)	544.50(1.00, 1156.50)	292.552	384.862	-461.763∼1046.867
**CHF-related symptoms**	**59.30 ± 11.98**	**-**	**-**	**-**	**-**	**45.001**	**6.1304**	**32.986~57.017**
**Kinesiophobia**	**44.39 ± 8.21**	**-**	**-**	**-**	**-**	**-55.741**	**8.737**	**-72.866~38.617**

### Factors associated with changes in PA over time during the postdischarge transition period

Table 2 showed the factors associated with changes in PA over time during the postdischarge transition period, as analyzed by the GEE multivariate model. Higher kinesiophobia (*Wald χ*^*2*^ = 10.417, *P* = 0.001) and CHF-related symptoms (*Wald χ*^*2*^ = 24.406, *P*<0.001) were significantly associated with a decrease in PA levels during the postdischarge transition period.

In addition, we used GEE models with time interactions to examine the impact of changes in CHF-related symptoms and kinesiophobia on PA during the postdischarge transition period. Compared with prehospitalization (baseline/T1), two weeks after discharge, CHF-related symptoms were significantly associated with a decrease in PA level (T2: *Wald χ*^*2*^ = 5.824, *P* = 0.021; T3: *Wald χ*^*2*^ = 5.314, *P* = 0.016).

**Table 2:** Factors associated with changes in PA over time based on the GEE analysis

Variables	β	SE	95%CI	*P*
Intercept	1645.068	1009.1055	-332.742∼3622.878	0.103
First diagnosis of CHF?				
Yes	0^a^	-	-	-
No	-178.810	173.818	-519.487∼161.867	0.304
NYHA Classification Grading				
II	0^a^	-	-	-
III	-854.942	533.5752	-1900.730∼190.846	0.109
CHF-related symptoms	**36.542**	**7.3968**	**22.045~51.040**	**<0.001**
T1*CHF-related symptoms	0^a^	-	-	-
T2*CHF-related symptoms	**-16.435**	**7.1293**	**-30.408~2.462**	**0.021**
T3*CHF-related symptoms	**-17.498**	**7.2504**	**-31.709~3.288**	**0.016**
T4*CHF-related symptoms	-12.164	7.9131	-27.674∼3.345	0.124
**Kinesiophobia**	**-32.888**	**10.1898**	**-52.859~12.916**	**0.001**
T1*Kinesiophobia	0^a^	-	-	-
T2*Kinesiophobia	6.884	9.7121	-12.151∼25.920	0.478
T3*Kinesiophobia	6.397	10.2670	-13.726∼26.520	0.533
T4*Kinesiophobia	3.111	10.5307	-17.529∼23.751	0.768

### The mediating effect of kinesiophobia on the relationship between CHF-related symptoms and PA

**Fig. 3:**
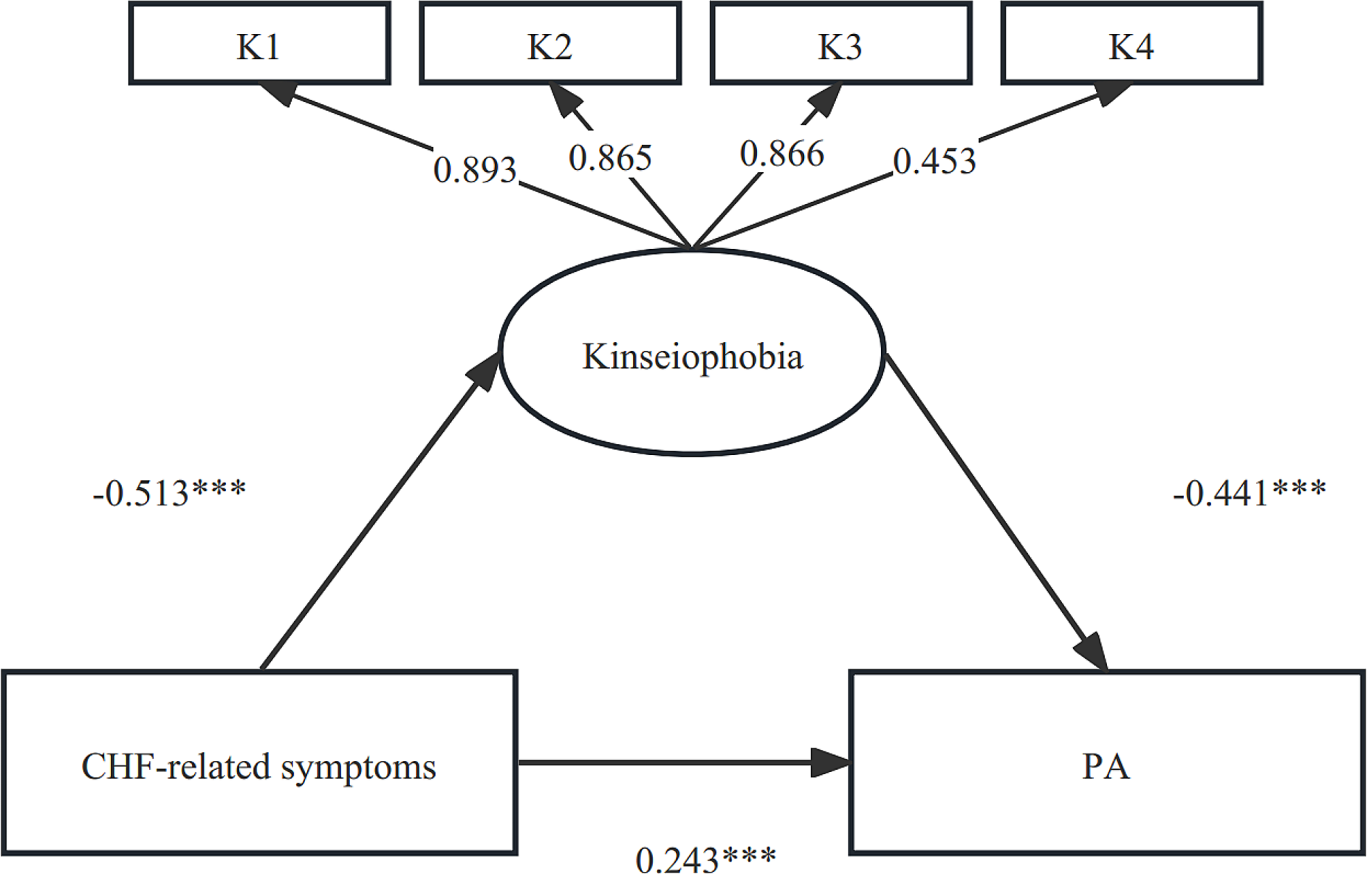
Direct and indirect effect of CHF-related symptoms on PA. Note: ***p value < 0.001

Path analysis revealed that the data fit the model adequately, with a model *χ*^*2*^ value of 23.04, *P* equal to 0.003, a ratio of chi-square to the degree of freedom equal to 2.88, a comparative fit index equal to 0.971, an adjusted goodness-of-fit index equal to 0.901, and a root-mean-square error of approximation equal to 0.038. CHF-related symptoms and kinesiophobia can be directly associated with PA in CHF patients (*β* = 18.053, *P* < 0.001) (*β*=-102.625, *P* < 0.001); the values of these effects were 0.243 and − 0.411, respectively; and CHF-related symptoms can directly associate with kinesiophobia (*β*=-0.153, *P* < 0.001), the value of this effect was − 0.153. In addition, CHF-related symptoms could indirectly affect PA through kinesiophobia. This effect was 0.211, indicating that kinesiophobia partially mediated the relationship between CHF-related symptoms and PA in CHF patients during the postdischarge transition period. The total model accounted for 46.48% of the variance in PA (Fig. 3).

## Discussion

Our results showed that patients with CHF had poor PA during the postdischarge transition period. The WHO standard for minimum active PA requires walking at least five days a week and engaging in moderate- to high-intensity physical activity, accumulating up to 600 MET min/week [26]. Overall, the PA of the subjects worsened over time in our study. It is undoubted that decreased PA occurs from preadmission to postdischarge for CHF patients within three months. However, transition points are vulnerable areas that contribute to high healthcare spending and lapses in quality and safety and are associated with increased hospitalization rates. A study showed that more than one-fifth of CHF patients were readmitted within 30 days, costing $17 billion [27]. Therefore, rehabilitation during this period is of crucial relevance. We need to explore the trajectory of PA changes and influencing factors during the postdischarge transition period to identify valuable intervention times and elements to promote the cardiac rehabilitation process, improve patients’ quality of life, and reduce their readmission rate.

Our study showed that the trajectory of CHF patients’ PA first decreased and then increased, with the lowest level occurring at two weeks after discharge. Within two weeks after discharge, the patients transferred from hospitals to their homes without adequate preparation for postdischarge life, making it difficult to adapt to the changes. However, our results showed that the PA gradually increased at two and three months after discharge. Patients’ adaptability and physical function improved as the transition period extended, increasing PA. Nevertheless, the chronic illness trajectory model suggests that CHF patients have long-term limitations on function with intermittent exacerbations; they meet by rescue treatment and often fail to return to nearly their prior functional status. This means that patients’ physical function and symptoms periodically deteriorate during each episode [28, 29]. Therefore, their PA three months after discharge did not return to the prehospitalization level. The findings suggested that healthcare professionals should assess dynamic PA levels and pay close attention to critical time points to promptly convey the value of PA and the correct approaches of PA to patients and their caregivers to help patients cope with challenges within two weeks after discharge and then gradually reduce the intervention intensity over time to promote the rational allocation of medical and nursing resources.

### Factors associated with PA

We found that more severe CHF-related symptoms and kinesiophobia were significantly associated with a decrease in PA during the postdischarge transition period. In line with the findings of previous cross-sectional study, patients often had obvious respiratory symptoms after activity, such as palpitations, chest tightness, and shortness of breath. These discomforts could seriously hinder enthusiasm for PA [30]. Additionally, due to irreversible pathological changes such as myocardial remodeling, the contraction and relaxation functions of the heart were always in an inefficient working state. Hence, patients’ symptoms were not significantly relieved at the early discharge stage. In addition, during the postdischarge transition period, patients not only face the transition from the medical environment to the family environment but also face situations involving low caregivers’ care knowledge and ability, which leads to slow recovery or even deterioration of disease [31]. As Seckin et al.’s study showed during follow-up, most patients’ self-reported symptom scores did not significantly change, but 25% of individuals reported worsening symptoms [32]. This was why the PA decreased along with CHF-related symptoms in our patients. These findings indicated that stabilizing CHF-related symptoms, especially in the early discharge stages, was crucial in cardiac rehabilitation.

Kinesiophobia is common in CHF patients and is a well-known barrier to the development of PA. A cross-sectional study demonstrated that approximately 31% of CHF patients reported moderate levels of kinesiophobia, whereas 24% of CHF patients reported high levels of kinesiophobia [33]. Khanna et al.’s study revealed a direct association between kinesiophobia and avoidance behavior [34]. Avoidance behavior is an overt or covert behavior that prevents or postpones an encounter with an aversive stimulus and usually implies activity restrictions, interference with valued life activities, and negative affect. A study by Brunetti et al. showed that a higher level of kinesiophobia correlated with lower PA, leading to an increasing possibility of intolerance, gradual weakening of muscle strength, and functional impairment in the body, which in turn exacerbated the severity of kinesiophobia [35]. Therefore, based on these findings, it was recommended that healthcare professionals help patients overcome kinesiophobia.

### The mediating effect of kinesiophobia on the relationship between CHF-related symptoms and PA

In this study, the improvement in CHF-related symptoms directly reduced kinesiophobia and enhanced PA in CHF patients. Furthermore, kinesiophobia mediated the relationship between CHF-related symptoms and PA, implying that the association between CHF-related symptoms and PA was partially through patients’ fear of PA. CHF-related symptoms are positively correlated with kinesiophobia. Qin et al.’s study showed that kinesiophobia gradually increased with the severity of HF symptoms [36]. According to the above results, kinesiophobia leads to maladaptive avoidance behavior, causing adverse health consequences such as physical inactivity. A previous study also confirmed that kinesiophobia was an essential mediator of PA in patients with cardiovascular disease [37], which was similar to our study results. These findings suggested that both CHF-related symptoms and kinesiophobia need to be identified and addressed as early as possible to avoid later deterioration of postdischarge transition PA. In clinical practice, healthcare professionals must perform pharmacological and nonpharmacological interventions to stabilize patients’ CHF-related symptoms, gradually reduce the discomfort experienced during activities, decrease patients’ kinesiophobia and increase their PA levels. These measurements may play a synergistic role in the nursing process and more effectively improve PA in CHF patients during the postdischarge transition period.

### Strengths and limitations

The main strength of the present study was that it was a longitudinal follow-up study in which cardiac rehabilitation effects were improved by measuring the change in PA over time and analyzing the associated factors. Path models were also used to construct theoretical models as part of efforts to increase our knowledge about the causal relationship between CHF-related symptoms and PA, as well as the mediating effects of kinesiophobia among CHF patients. As a limitation, the self-reported PA that was used in this study may be over-reported. Therefore, objective measurement tools in longitudinal studies should be pursued to ensure more accurate results. Moreover, as this investigation focused on the transition period, which included three months of follow-up, the PA might have exhibited different change trends over longer periods. Therefore, future studies could extend the follow-up time. Finally, we did not figure out what’s the most important signs or symptoms which correlated to the PA or the kinesiophobia. So, in the future study, we will conduct subgroup analysis to settle this question.

## Conclusion

During the postdischarge transition period, PA in CHF patients exhibited a low level and worsened over time. Patients with higher kinesiophobia levels and more severe CHF-related symptoms were at the most significant risk for decreasing PA. Moreover, kinesiophobia mediated the association between CHF-related symptoms and PA. Identifying these risk factors and the pathways associated with these factors are essential because at-risk individuals can be targeted for early assessment, and more effective interventions can be developed.

## Data Availability

The datasets used and analyzed during the current study are available from the corresponding author upon reasonable request.

## Abbreviations

CHF: Chronic heart failure
PA: Physical activity
GEE: Generalized estimating equation models
FA: Fear avoidance
STROBE: Strengthening the Reporting of Observational Studies in Epidemiology
NT-proBNP: N-terminal pro-B-type natriuretic peptide
BNP: B-type natriuretic peptide
NYHA: New York Heart Association
CHF-PRO: Patient-reported Outcome Measure for CHF
TSK-Heart: Tampa Scale for Kinesiophobia Heart
